# No evidence for the consistent effect of supplementary feeding on home range size in terrestrial mammals

**DOI:** 10.1098/rspb.2023.2889

**Published:** 2024-06-12

**Authors:** Astrid Olejarz, Tomasz Podgórski

**Affiliations:** ^1^ Department of Game Management and Wildlife Biology, Faculty of Forestry and Wood Sciences, Czech University of Life Sciences, Kamýcká 129, Prague 6-Suchdol, 165 00, Czech Republic; ^2^ Mammal Research Institute, Polish Academy of Sciences, Stoczek 1, 17-230 Białowieża, Poland

**Keywords:** artificial food, home range, meta-analysis, spatial behaviour, wildlife feeding

## Abstract

Food availability and distribution are key drivers of animal space use. Supplemental food provided by humans can be more abundant and predictable than natural resources. It is thus believed that supplementary feeding modifies the spatial behaviour of wildlife. Yet, such effects have not been tested quantitatively across species. Here, we analysed changes in home range size owing to supplementary feeding in 23 species of terrestrial mammals using a meta-analysis of 28 studies. Additionally, we investigated the moderating effect of factors related to (i) species biology (sex, body mass and taxonomic group), (ii) feeding regimen (duration, amount and purpose), and (iii) methods of data collection and analysis (source of data, estimator and spatial confinement). We found no consistent effect of supplementary feeding on changes in home range size. While an overall tendency of reduced home range was observed, moderators varied in the direction and strength of the trends. Our results suggest that multiple drivers and complex mechanisms of home range behaviour can make it insensitive to manipulation with supplementary feeding. The small number of available studies stands in contrast with the ubiquity and magnitude of supplementary feeding worldwide, highlighting a knowledge gap in our understanding of the effects of supplementary feeding on ranging behaviour.

## 1. Introduction

Spatial and temporal heterogeneity in the abundance of food resources is one of the key challenges animals face when navigating the environment. This variation shapes foraging decisions and space use of animals, which try to balance the energetic costs and benefits of acquiring food. Optimal foraging theory (OFT) posits that the most successful foraging strategy will minimize foraging costs to the benefit of increased fitness [1]. Resource-rich habitats offer high nutritional gains with a low-energetic cost of travel. Thus, animals’ home ranges are predicted to be smaller when resources are abundant [2]. Many species experience food provisioning through wildlife management practices [3], eco-tourism [4], recreational feeding [5,6], food waste mismanagement [7,8] and conservation efforts [9], potentially inducing changes in movement [10] and space use patterns [11]. In contrast to the general assumption that supplementary feeding leads to a reduction in the home range size, the literature provides ambiguous evidence, with some studies reporting a decrease [12–15], no change [16,17] or an increase [18,19]. The previous reviews on supplementary feeding [13,20,21] have qualitatively summarized various effects on wildlife but have not quantitatively addressed spatial behaviour.

While food resources are one of the key determinants of ranging behaviour, other factors like species biology also contribute to home range size. Home range size is positively related to body mass in mammals owing to the higher metabolic requirements of larger species [22–24]. Food is limited in space and time and, to fulfil metabolic requirements, larger animals have to increase foraging and travel costs. Supplemental food helps satisfy metabolic needs at lower foraging and travel costs and can thus be expected to cause a decrease in the home range size, potentially flattening out of the relationship between body size and home range size. Inter-specific differences in predator avoidance strategies [25], territorial behaviour [26,27] and social structure [23] can further modify the relationship between food availability and home range size across taxonomic groups. Energetic requirements differ between sexes and in many species, males have larger home ranges than females [28–31]. Female home ranges are further reduced during the rearing of offspring [32,33] owing to limited offspring mobility, protection from infanticide [34] and from predators [35]. Predictable and abundant food resources from supplementary feeding, particularly for females with offspring, could further enhance site fidelity. Males, on the other hand, can expand or maintain large home ranges to improve their reproductive success [36–39]. Those reproductive needs may outweigh the energy savings offered by supplementary feeding in shaping the home range [33]. Thus, we can expect a stronger spatial response to supplemental feeding in females.

Anthropogenic food resources are often more predictable, abundant and energy rich than natural resources [8] and they may provide a strong spatial signal inducing fidelity to resource-rich areas and, consequently, decline of home range [40]. However, those food resources should be consistently available for a long period of time to induce behavioural change. OFT assumes that, owing to their cognitive abilities, animals own complete knowledge of the spatio-temporal distribution of resources [41]. Yet, mammals continuously update their cognitive map to decide where to forage [42]. Only rich food patches leave a strong cognitive imprint on the spatial map of resources and can thus influence the decisions of where to move [31,43]. We can distinguish two different anthropogenic food resources: intentional and unintentional supplementary feeding. Intentional (subsidiary and artificial) feeding can be defined as placing natural or non-natural food into the environment to augment regular food sources [44] or attract animals [6]. The extent, intensity and form of wildlife feeding vary widely depending on its intended purpose. In game management, supplementary feeding is a deliberate tool to keep the target population stable or improve its performance, especially when natural food resources are scarce [20,21,45,46], or to mitigate damage to agricultural crops [47] and tree regeneration [48,49]. Thus, this type of feeding is often seasonal to address specific aims. The scale of game feeding is enormous: 2.8 trillion tons of bait are used annually in the USA [7] and 42 million USD worth of feed was provided to wildlife in Sweden in 2013 [50]. Unintentional feeding offers anthropogenic food sources (e.g. landfills, municipal and agricultural waste, and hunted game offal) but is not specifically targeted at feeding wildlife, and thus without pre-defined target species or a timeframe of feeding. This form of feeding is also very prevalent [7,51]. Up to 40% of all food products on Earth are wasted [7] and become a potential food resource for wildlife. Annually, tonnes of big game carrion in Europe and the USA serve as food for the large majority of vertebrate scavengers [7,47,52]. We can expect longer-lasting and more consistent unintentional feeding to produce a stronger habituation effect and thus have a stronger decreasing effect on the home range size. Food availability plays a crucial role in the spatial behaviour of animals and supplemental food can be expected to alter natural space use patterns. For example, the home range size of wood mouse in a low-quality habitat with supplementary feeding was smaller than without, and similar to high-quality deciduous woodland [36]. We can thus expect a stronger decrease in home range size when supplemental food is more abundant.

Home range is a fundamental outcome of animal movement [2], reflecting its ecology and spatial behaviour [42] and is one of the most commonly used metrics of space use [42]. Owing to the constant development of tracking technologies, analytical approaches and home range concepts [53–55], different methods exist to analyse home range data [56]. One common method is the geometric estimator, such as minimum convex polygon (MCP), which builds home range polygons by using the locations of an observed animal. Alternatively, probabilistic estimators, such as the kernel density estimator, calculate home range based on the frequency distribution of animal locations [57]. If animals preferentially use supplementary feeding sites and their surroundings, the kernel estimator should perform better in capturing this effect than the MCP, which does not account for the utilization distribution. Many species, particularly game, are kept temporarily in enclosures for ecological and economic reasons [58,59]. The enclosures can alter natural conditions by excluding predators and/or maintaining high population density and, consequently, high levels of intra-specific competition. Both of these factors may lead to smaller home ranges in terrestrial mammals [31,60,61]. We can expect that supplementary feeding in enclosures will lead to a stronger decrease in home range compared with free-ranging populations where additional variables (e.g. distribution of other vital resources, location of supplementary feeding sites and disturbances) may counterbalance the effect of feeding.

In this study, we collected the home range size of terrestrial mammals under supplementary feeding to examine its effect on space use. We created a meta-analysis of 28 studies from 23 species to test an overarching hypothesis of reduced home range size when supplementary feeding is present. Additionally, we investigated the modifying effect of three groups of factors related to (i) species biology (sex, body mass and taxonomic group), (ii) feeding regimen (duration, amount and purpose), and (iii) methods of data collection and analysis (source of spatial data, home range estimator and spatial confinement). The results are discussed in the context of wildlife conservation and game management.

## 2. Methods

### (a) Article search

We used Web of Science (WoS) and Scopus, two publisher-independent global citation databases, to identify published articles that analysed the effect of supplementary feeding on home range size in terrestrial mammals. On 21 March 2022, we created a Boolean operator, consisting of (i) ((‘Supplement* feed*’ OR ‘Artificial feeding’ OR ‘Food supplementation’ OR ‘Winter feeding’ OR ‘Bait* sites’ OR ‘Waste’ OR ‘Trash’ OR ‘Garbage’ OR ‘Recreational feeding’ OR ‘Dumps’ OR ‘Landfill’ OR ‘Refuse*’ OR ‘Feeding site’ OR ‘Supplemented food’ OR ‘Anthropogenic Resources’), (ii) AND (‘Home range*‘), and (iii) NOT (‘Bird’ OR ‘Amphibians’ OR ‘Fish’ OR ‘Reptiles’ OR ‘Radioactive’ OR ‘Human’)). In addition to the Boolean operator, we used an additional filter in the Scopus database by only allowing publications assigned to the subject area Agricultural and Biological Sciences and Environmental Sciences. We did not use platforms with grey literature for two reasons: (i) an advanced search with the help of the Boolean operator is only possible to a limited extent and (ii) platforms such as Open Grey and Google Scholar include unpublished and non-peer-reviewed research with results which could not be verified [62].

The search returned 191 hits in WoS and 162 hits in Scopus. To avoid duplications, the titles of both searches were compared in the package dplyr [63] in the R environment (v. 4.2.2 [64]), and duplications were removed from the Scopus table. In the end, this resulted in 259 scientific papers. In the next step, we conducted an abstract screening of all hits, excluding studies that did not focus on terrestrial mammals. After the abstract screening, 135 studies which were deemed potentially relevant underwent a second screening round of the full text. Only studies that reported home range sizes with and without supplementary feeding were selected, which provided a contrast of experimental treatment (supplementary feeding) and control group (no feeding). After the full-text screening, 24 scientific studies were selected, and we added four further publications that appeared in the reference lists of the 24 publications. In total, we used 28 publications for the meta-analysis (electronic supplementary material, figure S1).

### (b) Data extraction

We extracted the home range size, its standard deviation (s.d.) and sample size from each publication for the experimental group of animals with supplementary feeding and the control group without. All home range sizes were converted into square kilometres. If s.d. was not given in the publication, we either calculated the s.d. from the raw data or converted it from standard error (s.e.) or confidence interval (CI) [65]. For each home range comparison, we compiled information about the species, its taxonomic group (rodent, carnivore and ungulate), individual’s sex, supplementary food amount (limited or ad libitum) and feeding duration, spatial confinement (free-ranging or enclosure), source of spatial data (telemetry or capture-mark-recapture) and home range estimator (kernel density estimation (KDE) or MCP). Moreover, we divided the publications into intentional and unintentional feeding studies. We defined intentional feeding as supplementary food provided to a target species with a fixed timeframe and defined aim. Unintentional feeding, in contrast, was classified as supplementary food available from anthropogenic sources (e.g. landfills and municipality waste) but not specifically targeted at feeding wildlife, and thus without pre-defined target species or a timeframe of feeding. Therefore, we only analysed feeding duration for intentional feeding studies. We added body mass for each species from the panTHERIA database [66] to the collected dataset.

### (c) Statistical analysis

To measure the change of the home range size from no-feeding to feeding treatment, we used the Hedges’ *g* estimator of the effect size [67]. For each study, we calculated the Hedges’ *g* and its variance with the escalc() function of the metafor package in R [68]. Negative values of the Hedges’ *g* represent the decrease in the home range size owing to feeding. Some publications provided multiple home range comparisons across seasons, sex and areas which contributed to a final total of 64 effect sizes from 28 studies. The publication bias within the collected dataset was visually examined with a funnel plot [69] and tested with Egger’s regression [70]. Meta-analytic mixed-effects models, fitted with the function rma.rm of the metafor package, were used to examine the effects of supplementary feeding on home range size. An intercept-only model was used to determine the mean effect size across the entire dataset. The proportion of heterogeneity relative to sampling error was quantified with the *I*^2^ statistic [71], from the intercept-only model. We created a random effect variable, ‘group within study’, which combined study identity and within-study grouping levels (e.g. areas, sex and seasons) in one unique identifier and thus accounted for between and within study variance. We then fitted nine separate meta-analytic mixed effects models to examine the effect of different moderators (i.e. sex, body mass, taxonomic group, food amount, feeding purpose, feeding duration, spatial confinement, source of spatial data and home range estimator) (electronic supplementary material, tables S1 and S2). We created a forest plot for all nine predictors using the modelplot function of the modelsummary package [72].

## 3. Results

**Figure 1.**
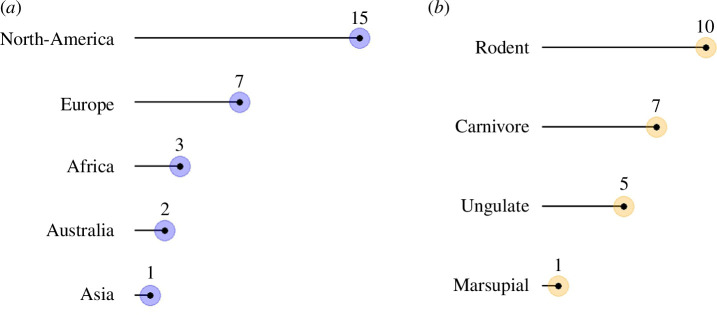
Distribution of studies across continents and species across taxonomic groups in the meta-analysis. (*a*) Number of studies per continent and (*b*) number of species per taxonomic group.

**Figure 2.**
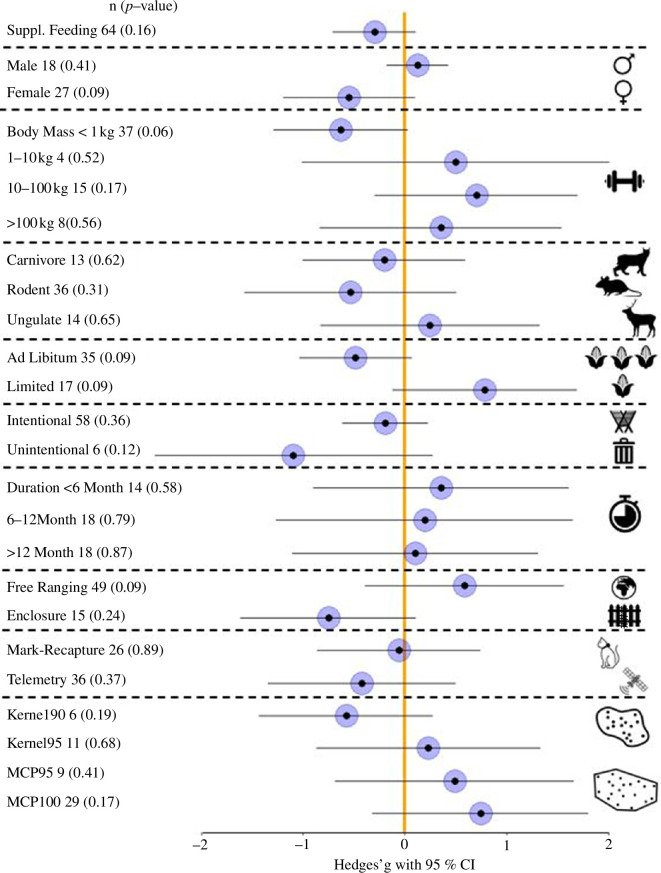
Response of home range size to supplementary feeding (sample size, *p*‐value, Hedges’ *g* estimate and its 95% CI) across all effects and for nine moderators: sex, body mass, taxonomic group, food availability, feeding purpose, feeding duration, presence of enclosure, source of spatial data and home range estimator. Positive values of Hedges’ *g* indicate an increase in home range size during supplementary feeding and negative values indicate a decrease.

In total, 28 studies originating from 24 countries and 23 species met our criteria for meta-analysis (table 1, figure 1). From those studies, we extracted 64 effects, which were symmetrically spread in the funnel plot (electronic supplementary material, figure S2), indicating no publication bias in the dataset. This was confirmed by the insignificant result of the Egger's test (*e* = 0.541, *p* = 0.522). Across the studies, we found moderate heterogeneity (*I*^2^ = 33.68%). The overall effect of supplementary feeding on the home range size was negligible (*g* = −0.297, 95% CI = −0.708 to 0.113, *p* = 0.1553, *n* = 64 effects; figure 2; electronic supplementary material, table S2).

**Table 1.** Studies used in the meta-analysis examining changes in the home range size of terrestrial mammals in response to supplementary feeding.

references	class	species	data
Akbar & Gorman [36]	rodent	*Apodemus sylvaticus*	telemetry
Broughton & Dickman [73]	marsupial	*Isoodon obesulus*	mark-recapture
Campbell *et al*. [74]	ungulate	*Odocoileus virginianus*	telemetry
Cooper *et al*. [75]	ungulate	*Odocoileus virginianus*	telemetry
Desy *et al*. [25]	rodent	*Microtus ochrogaster*	telemetry
Dickmann [76]	rodent	*Antechinus stuartii*	mark-recapture
Festerer *et al*. [17]	carnivore	*Ursus americanus*	telemetry
Gilchrist & Otali [77]	carnivore	*Mungos mungo*	telemetry
Grenier *et al*. [78]	ungulate	*Odocoileus virginianus*	telemetry
Hall *et al*. 1998 [79]	rodent	*Peromyscus truei*	telemetry
Haspel & Morrison [80]	carnivore	*Felis catus*	observation
Hidalgo-Mihart *et al*. [81]	carnivore	*Canis latrans*	telemetry
Lacki *et al*. [82]	rodent	*Tamias striatus*	boundary strip
Lopez Bao *et al*. [83]	carnivore	*Lynx pardinus*	telemetry
McRae *et al*. [84]	ungulate	*Sus scrofa*	telemetry
Mondajem & Perrin [85]	rodent	*Mastomys natalensis*	mark-recapture
Morris *et al*. [86]	rodent	*Sigmodon hispidus*	telemetry
Mysterud *et al*. [87]	ungulate	*Cervus elaphus*	telemetry
Ranc *et al*. [88]	ungulate	*Capreolus capreolus*	telemetry
Reinecke *et al*. [89]	ungulate	*Cervus elaphus*	telemetry
Rotem *et al*. [90]	carnivore	*Canis aureus*	telemetry
Schoepf *et al*. [91]	rodent	*Rhabdomys pumilio*	telemetry
Sulok *et al*. [92]	rodent	*Sigmodon hispidus*	mark-recapture
Teferi & Millar [93]	rodent	*Peromyscus maniculatus*	mark-recapture
Todorov *et al*. [94]	carnivore	*Ursus arctos*	telemetry
Van Beest *et al*. 2010 [49]	ungulate	*Alces alces*	telemetry
Webb *et al*. [19]	ungulate	*Odocoileus virginianus*	telemetry
Young *et al*. [32]	carnivore	*Canis latrans*	telemetry

### (a) Species biology

We did not find consistent effects of biological moderators (sex, body mass or taxonomic group) on the relationship between supplementary feeding and home range size (figure 2). Opposite tendencies were observed between sexes. Females showed a relatively strong reduction in the home range size owing to supplementary feeding (*g* =−0.55, 95% CI = −1.2 to 0.1) while a slight increase (*g* = 0.13, 95% CI = −0.18 to 0.43) was observed in males. Species body mass showed no consistent effect on changes in home range in response to feeding, despite a 20 000-fold range of this parameter in the dataset (0.02–461.9 kg). Only species with a body mass below 1 kg (91% rodents and 9% marsupial) showed a strong tendency of reduced home range size (*g* = −0.63, 95% CI = −1.29 to 0.03). Heavier species showed the opposite trend of increased home range (figure 2). The taxonomic groups showed a mixed response to supplementary feeding, with home range size tending to decrease in rodents (*g* =−0.54, 95% CI = −1.57 to 0.5) and carnivores (*g* =−0.2, 95% CI = −1.0 to 0.6) while increasing in ungulates (*g* =0.25, 95% CI = −0.83 to 1.32).

### (b) Feeding regimen

We did not find consistent effects of supplementary feeding on home range size in relation to food amount, feeding purpose and duration (figure 2). Animals with access to unlimited (ad libitum) food resources showed a relatively strong tendency to decrease in home range size (*g* =−0.48, 95% CI = −1.04 to 0.07), while those that received limited supplementary food tended to strongly increase the home range size (*g* =0.79, 95% CI = −0.12 to 1.69). Duration of intentional supplementary feeding averaged 16 months (min–max: 1–84). Despite the large time range, home range size showed little change in response to feeding duration. Feeding duration showed a consistently positive yet weak effect on the home range size, with *g* ranging from 0.1 to 0.36 for the feeding duration of >12 months and <6 months, respectively. The purpose of feeding did not affect the way animals responded to food provisioning but it modified the strength of the response. Both intentional and unintentional feeding induced a tendency of reduction in the home range size with a notably stronger effect of unintentional feeding. In fact, unintentional feeding had the strongest effect (*g* = −1.1, CI = −2.48 to 0.28) of all moderators tested (*g* < 0.8) (figure 2; electronic supplementary material, table S2).

### (c) Methods of data collection and analysis

Contrasting trends were observed for the spatial confinement of the studied animals. Animals in enclosures tended to reduce home range size under supplementary feeding (*g* =−0.75, 95% CI = –1.61 to 0.11), whereas free-ranging ones tended to increase home range size (*g* =0.58, 95% CI = −0.4 to 1.56). Most of the studies (76%) were conducted on free-ranging animals and all enclosure studies were conducted on rodents. Methods of spatial data collection did not change the relationship between supplementary feeding and home range size, though a stronger signal for reduced home range was detected for telemetry-derived data (*g* =−0.42, 95% CI = −1.34 to 0.5) compared with mark-recapture data, which showed virtually no effect (*g* =−0.06, 95% CI = −0.86 to 0.74) (figure 2). Across home range estimators, only kernel 90 showed a negative tendency of reduced home range under feeding (*g* =−0.58, 95% CI = −1.43 to 60.28). All of the other estimators showed positive trends of varying strength, with the strongest signal for MCP 100 (*g* = 0.74, 95% CI = −0.32 to 1.81) (figure 2).

## 4. Discussion

Our literature search yielded 28 studies (64 effects) containing proper control of feeding experiments. This relatively small sample stands in glaring contrast with the ubiquity and magnitude of supplementary feeding worldwide and highlights the need for further research on the effects of supplementary feeding on movement and space use. Nevertheless, the results of this meta-analysis provided new and unexpected insights. Contrary to the assumption made by OFT, the effect of higher food availability offered by supplementary feeding on the reduction in the home range size was inconclusive. While a negative trend towards smaller home ranges under supplementary feeding was observed, our dataset provided weak evidence (*p* = 0.16) against the null hypothesis of no effect. It should be noted that home range behaviour is shaped not only by the availability of food but also by other resources, such as predation risk, population density and social interactions, which OFT does not account for. The predation strongly influences foraging behaviour as animals make foraging decisions in relation to predation risk to maximize biological fitness [95–98]. Trade-off between food and safety can force animals to forage over larger areas if the presence of predators compromises foraging opportunities [99]. Rearing of offspring can also strongly impact home range size. Around parturition, female white-tailed deer [100,101] and wild boar [102] reduce their movements and home range size. Food availability can interact with the distribution of other key resources to influence the home range size. In semi-arid environments, density of and proximity to waterholes has been shown to be inversely related to home range size in African elephants [103] and southern mule deer [104].

### (a) Species biology

Factors related to species biology, feeding regime and methods of data collection and analysis had varying moderating effects, in size and direction, on the relationship between supplementary feeding and home range size. Space used by animals is expected to increase with increasing body mass [22,105]. Our results did not show a consistent effect of body mass on the relationship between supplementary feeding and home range size across a wide range of body sizes considered (0.02–461.9 kg). Species with body mass below 1 kg (91% of which were rodents) tended to show a relatively strong negative response when supplementary food was provided. There was a 6% probability of our result arising by chance, assuming no effect in this weight class, which provided moderate evidence for the effect. Heavier species, on the other hand, showed positive trends with large uncertainty and weak support against null hypothesis of no effect (*p*-values from 0.17 to 0.56). Small mammals have higher energy expenditure relative to their body size than large mammals as they spend more energy regulating body temperature [24,106]. It is possible that energetic constraints of small mammals make them sensitive to manipulation of food resources and easier to induce spatial response. Larger and more energetically robust species, on the other hand, may require greater amounts and/or more nutritionally rich food for the spatial response to manifest. Besides the relationship between body mass and home range size, space use patterns are also influenced by sexual differences [107]. Consistent with our expectation, females showed a strong tendency to decreased home range size after supplementary feeding and a moderate concordance with the null hypothesis of no effect (*p* = 0.09), providing some support for the effect. Males contrasted with a negligible change in home range size. Differences in mating strategies can lead to contrasting space use patterns in males and females. According to the range size hypothesis [37], superior males in promiscuous or polygynous mating systems roam widely for multiple mates [36,38,39]. Our results indicate that male movement decisions can be driven by reproductive needs rather than energy savings offered by supplementary feeding. In contrast, predictable and abundant food resources offered by supplementary feeding can enhance further site fidelity in females, particularly those with offspring, which are already constrained by energetic requirements of gestation or lactation, immobility of offspring and the need to protect them from predation [34,35].

Response in home range size owing to supplementary food varied in three taxonomic groups. The marginal tendency of home range size decrease was identified for carnivores and rodents, while a weak trend of home range increase was observed in ungulates. However, those results were in high concordance with the null hypothesis of no effects of supplementary feeding on home range size across taxonomic groups (*p*-values from 0.31 to 0.65) and thus provided poor evidence for the actual differences. An adequate size of a home range in carnivores is not only determined by the available food resources but also by social organization [23]. Many carnivore species show pronounced territorial behaviour [26,27] and maintain their home range size in relation to population density [108,109]. This fairly rigid socio-spatial population structure can allow only limited changes in home range size in response to feeding [32]. Additionally, carnivores rely on mobile and patchily distributed food, which requires large home ranges [110,111]. Spatially predictable resources offered by supplementary food could partly relax the requirement of large home ranges. A declining trend in home range size was also observed in rodents but this pattern could be triggered by a different behavioural mechanism compared to carnivores. Rodents, which are prey species to many other animal groups, adopt the strategy of staying close to burrows and reducing their home range size when predation risk is high [25]. Supplemental feeding could potentially enhance movement restriction in the face of high predation risk, but we were not able to test this effect with our data. Ungulates, on the other hand, tended to increase home range size under supplementary feeding. It has been shown in white-tailed deer [19] and roe deer [88] that the location of feeding stations at the periphery of the core range can lead to home range shift and increase in range size. Although this effect is not necessarily specific to ungulates, it could be responsible for the increase in home range size observed in 5 out of 9 studies on ungulates that we analysed. In all considered taxonomic groups, the potential effects of supplemental food could have been balanced out by other drivers of home range behaviour, such as predation avoidance, social interactions and other resources.

### (b) Feeding regimen

Unexpectedly, opposing trends in the direction of change in the home range size were observed between studies providing a limited and unlimited (ad libitum) amount of food. In experiments with limited feeding, food was delivered according to a specific protocol at fixed quantities and time intervals. However, we were not able to compile information on the exact amount of food provided in relation to species nutritional demands and quantify the degree of food limitation. In unlimited feeding experiments, in contrast, food was available all the time and topped up as depleted. Animals receiving unlimited food tended to decrease their home range size while the opposite trend was observed when the amount of supplemental food was limited. Both estimates had a 9% probability of being obtained by chance, assuming no differences in home range size depending on the amount of supplementary food, which provided some evidence for the effects. It is possible that food limitation provided a too-weak signal to induce changes in home range size. In free-ranging cats, for example, only continuous supplementary feeding can efficiently reduce home range size in an unproductive habitat [80].

When feeding intentionally, it is usually possible to track the duration of supplementary feeding, while the time at which wild animals start to use unintentional anthropogenic food sources is usually unknown. In our meta-analysis, unintentional feeding induced a much stronger negative response in home range size compared with intentional feeding. All studies with unintentional feeding reported a substantial reduction in the home range size when food was available [77,81,90,112], while studies with intentional feeding reported no consistent response in home range size change. The strong spatial response of wildlife after the closure of unintentional feeding spots can be attributed to the cognitive abilities of mammals [31,42,43]. For example, spotted hyenas preferentially used areas around human waste pits which constituted a primary food source. After closure of the pits, home range size of the spotted hyenas increased [112]. Golden jackals maintained smaller home ranges near villages compared with natural areas, supposedly in response to availability of anthropogenic food [90].

Most studies with unintentional feeding did not provide an exact timeframe of food provisioning and therefore only studies with intentional feeding were included in the analysis of the effect of feeding duration. Feeding durations covered a wide range of timespans (from 1 to 84 months), but we found a very weak trend of increasing home range size regardless of whether feeding was short (<6 months), medium (6–12 months) or long terms (>12 months). Additionally, this data provided no evidence against the null hypothesis of no effect of supplementary feeding depending on its duration (*p*-values from 0.58 to 0.87). Intentional feeding is often used to prevent damage to agricultural fields by abundant game species, such as deer and wild boar [113]. As feeding duration in this case is often limited to one season [114,115], the anticipated change of ranging behaviour through supplementary feeding might not occur, as demonstrated in our meta-analysis.

### (c) Methods of data collection and analysis

Twenty-four per cent of the studies were conducted in enclosures, exclusively on rodents. The enclosures were usually large enough to not constrain movement (i.e. larger than home range size) but could exclude predators or sustain high individual densities and high levels of intra-specific competition. Free-ranging mammals showed a tendency to increase home range size with supplementary feeding, while the opposite trend was observed in enclosures. Free-ranging data provided some evidence for the observed difference (*p* = 0.09), while data from enclosures was concordant with the null hypothesis of no effect (*p* = 0.24). One possible explanation might be that, in the open settings, feeding sites were located on the periphery of an animal’s home range, causing additional movement [19,32]. Supplementary feeding in enclosures tended to lead to smaller home ranges. If enclosures exclude predators, prey can become less vigilant [116] and feel less pressure to seek out and explore different habitats to hide from predators [117]. Besides removing predation risk, enclosures can also increase intra-specific competition, as there is no way to migrate out of the enclosure. High intra-specific competition for the habitat can force animals to keep the home range small and to increase it when intra-specific competitors are lacking [31,60,61].

Home range reflects an animal’s ecology and behaviour in space [42] and estimations of home range area are widely applied in animal ecology [118]. While almost any type of animal location data can be used to calculate home range, higher temporal granularity and spatial accuracy will yield more precise and biologically relevant estimates. We have identified two major types of spatial data used in the studies we examined: capture-mark-recapture and telemetry (radio and GPS). The former is typically of much lower spatio-temporal resolution than the latter, which can result in less precise estimation of home range size. Yet, we found that the type of spatial data used to calculate home range size did not consistently affect its response to supplementary feeding. While home ranges based on capture-mark-recapture data showed no response to supplementary feeding, telemetry-based home ranges showed a decreasing trend in response to feeding. In our meta-analysis, we included two broad categories of home range estimators, historically older MCP and more modern KDE. Differences in assumptions, calculations and interpretations between the estimators may have led to different trends in our meta-analysis results. Home ranges computed with kernel 90 showed a relatively strong decline under supplementary feeding, whereas all the other estimators tended to show a positive effect, with the strongest for MCP 100. MCP is frequently used in animal studies because it is easy to compute and compare [119,120]. Yet, it is sensitive to outliers, sample size and spatial resolution, which can create estimates biased upwards and does not reflect intensity of use. Kernel estimators, on the other hand, are relatively unbiased and account for centres of activity [53]. Thus, kernel-based methods are better suited to study the effects of resource distribution on space use. In our meta-analysis, only isopleth 90 of the kernel, representing the innermost part of the home range of all estimators considered, showed a trend similar in strength and direction to the overall effect of supplementary feeding on home range size. Interestingly, Borger *et al*. [53] found kernel 90 to be less biased and more accurate than outer kernel isopleths and MCP estimators.

## 5. Conclusion

Contrary to the common belief and the prediction of OFT that supplementary feeding would reduce home range, we did not detect a consistent effect with our meta-analysis. While an overall tendency of reduced home range was observed, the effect was not consistent across the available studies and the uncertainty around the estimate indicated the possibility of no effect. Moreover, moderators varied in the direction and strength of the trends, highlighting inconsistencies in the effects of supplementary feeding on home range size depending on species biology, feeding management and home range estimation methods. We conclude that home range size is resistant to manipulation with supplementary feeding owing to a multitude of drivers and complex mechanisms of home range behaviour. Despite the widespread practice of wildlife feeding, our literature research shows that only a small amount of data exists that examines the effect of supplementary feeding on the spatial behaviour of terrestrial mammals. More comprehensive research and clear policies are needed to better understand and manage the effects of supplementary feeding on spatial behaviour. In wildlife management, it is recommended to weigh the economic, health and ecological risks before providing supplementary food. In the case of unintentional feeding through food waste, initial steps have been taken by the European Union (EU). In 2016, an EU Platform on Food Losses and Food Waste was created. Combining a group of EU institutions, experts and international organizations, the platform aims to prevent food waste and to share best practices. The European Commission has set the target of reducing food waste in food manufacture and processing by up to 10% and by up to 30% in households by 2030.

## Data Availability

Data and R Scripts are available from the Dryad Digital Repository [121]. Electronic supplementary material is available online [122].
